# Genetics of environmental sensitivity to psychiatric and neurodevelopmental phenotypes: evidence from GWAS of monozygotic twins

**DOI:** 10.21203/rs.3.rs-4333635/v1

**Published:** 2024-05-02

**Authors:** Elham Assary, Jonathan Coleman, Gibran Hemani, Margot van Der Veijer, Laurence Howe, Teemu Palviainen, Katrina Grasby, Rafael Ahlskog, Marianne Nygaard, Rosa Cheesman, Kai Lim, Chandra Reynolds, Juan Ordoñana, Lucia Colodro-Conde, Scott Gordon, Juan Madrid-Valero, Anbupalam Thalamuthu, Jouke-Jan Hottenga, Jonas Mengel-From, Nicola J. Armstrong, Perminder Sachdev, Teresa Lee, Henry Brodaty, Julian Trollor, Margaret Wright, David Ames, Vibeke Catts, Antti Latvala, Eero Vuoksimaa, Travis Mallard, K Harden, Elliot Tucker-Drob, Sven Oskarsson, Christopher Hammond, Kaare Christensen, Mark Taylor, Sebastian Lundström, Henrik Larsson, Robert Karlsson, Nancy Pedersen, Karen Mather, Sarah Medland, D Boomsma, Nicholas Martin, Robert Plomin, Meike Bartels, Paul Lichtenstein, Jaakko Kaprio, Thalia Eley, Neil Davies, Patricia Munroe, Robert Keers

**Affiliations:** Kings College London; Institute of Psychiatry, Psychology, and Neuroscience, King's College London; University of Bristol; Vrije Universiteit Amsterdam; University of Bristol; Institute for Molecular Medicine Finland FIMM, University of Helsinki, Helsinki; QIMR Berghofer Medical Research Institute; Uppsala University; Danish Twin Registry; University of Oslo; King's College London; University of Colorado Boulder; University of Murcia; QIMR Berghofer Medical Research Institute; QIMR Berghofer Medical Research Institute; University of Murcia; University of New South Wales; Vrije Universiteit; University of Southern Denmark; Curtin University; University of New South Wales; University of New South Wales; Centre for Healthy Brain Ageing; University of New South Wales; The University of Queensland; University of Melbourne; University of New South Wales; University of Helsinki; University of Helsinki; Massachusetts General Hospital; University of Texas at Austin; The University of Texas at Austin; Uppsala University; King's College London; University of Southern Denmark; Karolinska Institutet; University of Gothenburg; Dummy; Karolinska Institutet; Karolinska Institute; Centre for Healthy Brain Ageing, Psychiatry, University of New South Wales (UNSW); QIMR Berghofer Medical Research Institute; Vrije Universiteit Amsterdam, The Netherlands; QIMR Berghofer Medical Research Institute; Institute of Psychiatry, Psychology and Neuroscience, King's College London, London; VU University Amsterdam; Karolinska Institute; University of Helsinki; King's College London; University College London; Queen Mary University of London; Queen Mary University of London

**Keywords:** vQTLs, variance effects, gene-environmental interaction, environmental sensitivity

## Abstract

Individual sensitivity to environmental exposures may be genetically influenced. This genotype-by-environment interplay implies differences in phenotypic variance across genotypes. However, environmental sensitivity genetic variants have proven challenging to detect. GWAS of monozygotic twin differences is a family-based variance analysis method, which is more robust to systemic biases that impact population-based methods. We combined data from up to 21,792 monozygotic twins (10,896 pairs) from 11 studies to conduct the largest GWAS meta-analysis of monozygotic phenotypic differences in children and adolescents/adults for seven psychiatric and neurodevelopmental phenotypes: attention deficit hyperactivity disorder (ADHD) symptoms, autistic traits, anxiety and depression symptoms, psychotic-like experiences, neuroticism, and wellbeing. The SNP-heritability of variance in these phenotypes were estimated (h2: 0% to 18%), but were imprecise. We identified a total of 13 genome-wide significant associations (SNP, gene, and gene-set), including genes related to stress-reactivity for depression, growth factor-related genes for autistic traits and catecholamine uptake-related genes for psychotic-like experiences. Monozygotic twins are an important new source of evidence about the genetics of environmental sensitivity.

## Introduction

Individual sensitivity to environmental exposures may be genetically influenced. This genotype-by-environment interplay implies differences in phenotypic variance across genotypes^1,2^. However, environmental sensitivity genetic variants have proven challenging to detect. GWAS of monozygotic twin differences is a family-based variance analysis method, which is more robust to systemic biases that impact population-based methods^3,45–8^. We combined data from up to 21,792 monozygotic twins (10,896 pairs) from 11 studies to conduct the largest GWAS meta-analysis of monozygotic phenotypic differences in children and adolescents/adults for seven psychiatric and neurodevelopmental phenotypes: attention deficit hyperactivity disorder (ADHD) symptoms, autistic traits, anxiety and depression symptoms, psychotic-like experiences, neuroticism, and wellbeing. The SNP-heritability of variance in these phenotypes were estimated (h^2^: 0–18%), but were imprecise. We identified a total of 13 genome-wide significant associations (SNP, gene, and gene-set), including genes related to stress-reactivity for depression, growth factor-related genes for autistic traits and catecholamine uptake-related genes for psychotic-like experiences. Monozygotic twins can provide important evidence about the genetics of environmental sensitivity.

## Main

Complex phenotypes are likely to be affected by genetic and environmental factors and interactions between them. Interactions between genetic variants and the environment will increase phenotypic variance. Genome-wide variance quantitative trait locus (vQTL) analysis^5,9^ aims to discover genetic variants associated with phenotypic variance. In contrast to most GWAS, which estimate associations of genetic variants and the phenotypic *means*, vQTL GWAS estimate the association of genetic variants and phenotypic *variance*. Phenotypic variance may be affected not only by gene-environment interactions^1^, but also by selection^3^, phantom vQTLs^4^, and epistasis^5^. Furthermore, commonly used population-based methods for estimating vQTLs using unrelated individuals suffer from variance inflation^6^, bias due to insufficient correction of demographic and indirect genetic effects^7^, and unstable test statistics when tested loci are in linkage disequilibrium with additive effects^8^. GWAS of monozygotic (MZ) twin differences provides an alternative, family-based approach to estimate vQTLs, which is less susceptible to these sources of bias^10^ and, therefore, provides more reliable evidence of genetic sensitivity to the environment.

**Figure 1:**
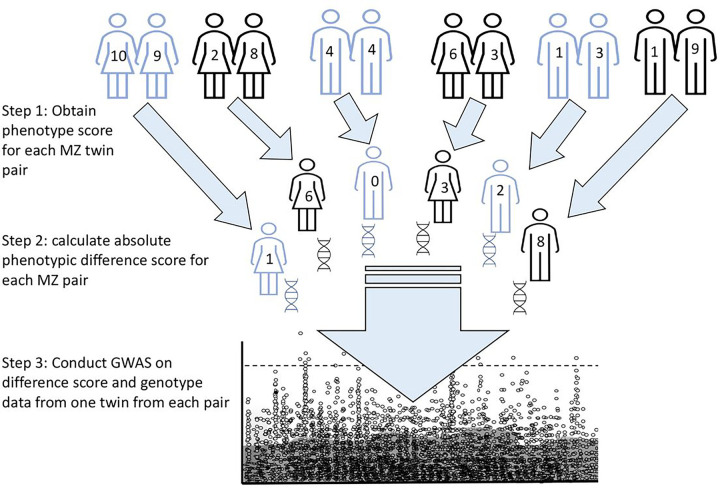
GWAS of MZ differences for estimating variance quantitative trait loci (vQTLs). GWAS of MZ difference approach. The analyses are conducted in three main steps: First, quantitative phenotype value is obtained for a population of MZ twin pairs. Second, the absolute phenotypic difference score is calculated for each MZ pair; the score reflects phenotypic differences due to environmental effects, as the environment makes genetically identical twins diverge phenotypically. The absolute phenotypic difference was corrected for age, sex, ten genetic principal components, and any study-specific covariates. The residuals were standardised and rank-transformed. Third, GWAS of MZ differences score is conducted, using phenotypic difference score for one twin from each pair and their genotype data, so the sample comprises unrelated individuals.

MZ twins are genetically nearly identical. Therefore, within-pair phenotypic differences are likely due to chance or the environment^11^. Somatic mutations may also play a role in MZ differences for chromosomal and rare diseases^12^ but are unlikely to play a systematic role in common and polygenic complex traits^13^. Though all MZ twin pairs have the same degree of genetic similarity, they have varying degrees of phenotypic similarity. In other words, within-pair phenotypic differences (variance) may vary by genotype across MZ twins. Thus, GWAS of MZ phenotypic differences can identify the loci associated with phenotypic variance (see Fig. 1 for a schematic representation of the GWAS of MZ differences). Since within-pair differences are due to the environment and unlikely to contain epistatic effects, the associations are a more robust measure of genetic contribution to environmental sensitivity than population-based approaches.

Though this approach has been advocated for understanding the genetics of environmental sensitivity^10^, the requirements for a large sample of monozygotic twins have been a major impediment to progress in this field. Here, we report findings from a GWAS-meta-analysis of MZ differences for seven psychological phenotypes, using data from up to 21,792 MZ twins (10,896 pairs) from 11 studies. This is the largest GWAS conducted on MZ twin differences to date, representing an order of magnitude more participants than two previous MZ twin differences GWAS^14,15^. We conducted meta-analyses separately for children and adolescents/adults and identified 13 genome-wide significant associations across our phenotypes. We estimated, for the first time, the SNP-heritability of environmental sensitivity to mental health phenotypes (adolescent ADHD = 0.18, se = 0.11; child ADHD = 0.04, se = 0.06; adult autistic traits = 0.09, se = 0.15; depression = 0.03, se = 0.09). We also found that higher genetic liability to depression and anxiety was associated with greater environmental sensitivity to depression in adults.

**Figure 2:**
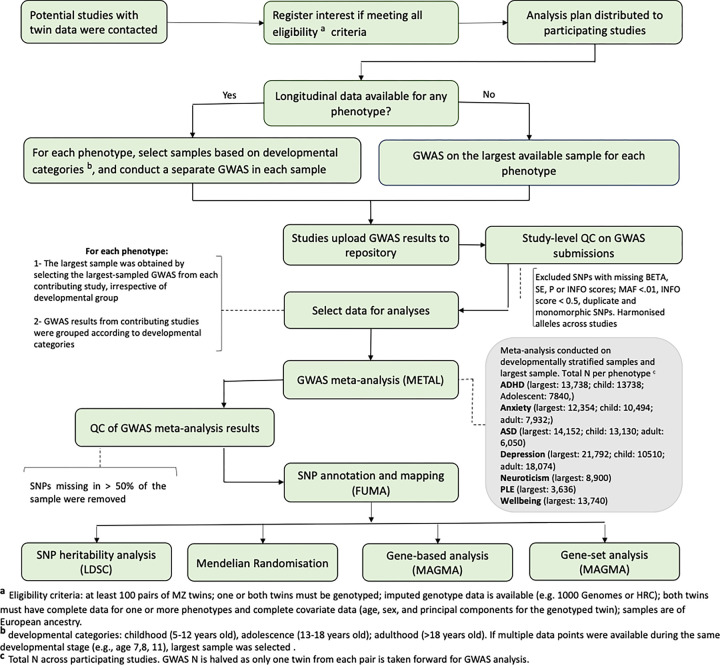
Flow chart of the current study

The empirical analyses included GWAS meta-analysis, gene-based and gene-set analysis, and Mendelian randomisation analyses (see Fig. 2 for a flow chart for the study). Contributing studies were asked to conduct GWAS of MZ differences separately for child, adolescent and adult samples if repeated measures across lifespan were available. The study-level GWAS results were subjected to QC and harmonisation (see **Supplementary Information section 2.2)** using EasyQC (v. 23.8)^16^, then meta-analysed using inverse-variance weighted fixed effect meta-analysis method in METAL (2011 release)^17^. GWAS meta-analyses were conducted using the largest available sample from each study and separately in developmentally- stratified samples (see methods). GWAS meta-analysis results were QC’d (see methods) and used for gene-based and gene-set analyses using MAGMA (v1.08) in FUMA web application (v1.5.2)^18^, and SNP-heritability analyses using LDSC (v1.0.1)^19^. We used Mendelian Randomisation to estimate the effects of psychological phenotypes (as reflected in GWAS associations with means) on phenotypic variance (see methods).

Table 1 shows descriptive statistics of the GWAS meta-analysis samples per phenotype. The Supplementary Information (**Section 1.1 and AllStudy_phenotype.xls**) presents participating studies and descriptive statistics. Depression symptoms had the largest sample, with 21,792 MZ twins from 11 studies, and psychotic-like experiences had the smallest, with 3,636 twins from two cohorts. Mean MZ correlations across studies ranged between r = 0.31 for wellbeing and r = 0.71 for child ADHD. Overall, the within-twin MZ correlations tended to be lower in adult samples than in child samples.

**Figure 3:**
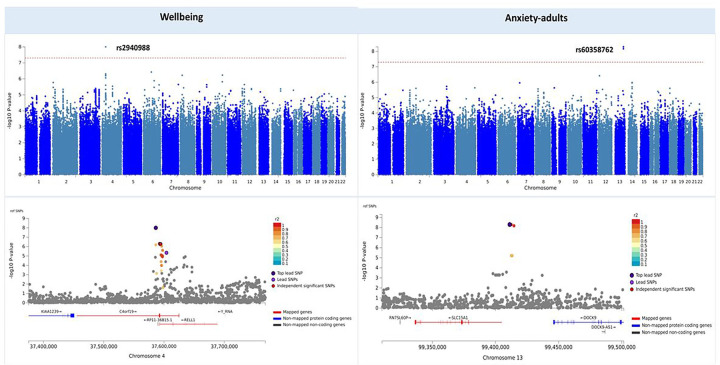
Manhattan and regional plots of genome-wide significant SNPs

Meta-analyses per phenotype identified a total of two genome-wide significant variants (Table 2), one associated with variability in wellbeing (rs2940988, *p* = 9.93e-9), located in the intronic region of the protein-coding, chromosome 4 open reading frame 19 (*C4orf19*) gene, and one variant (rs60358762, *p* = 5.07e-9) associated with variance in anxiety symptoms in adults, located in the intergenic region of the protein-coding *SLC15A1* gene on chromosome 13. Manhattan, QQ plots and genomic regions of these genome-wide significant variants are presented in Fig. 3. No variants were genome-wide significantly associated with variance in the other phenotypes (see **Table S4** for top SNPs for each phenotype). See Supplementary Information (**Figures S7-S11**) for Manhattan and QQ plots for all phenotypes.

The MAGMA gene-based analysis (methods) found several genes associated with phenotypic variability; however, only two associations passed the Bonferroni correction for multiple testing (Table 2). The Patched 1 (*PTCH1*) gene was associated (*p* = 1.80e-06) with variance in depression. The chromosome 15 open reading frame 38 (*C15orf38*) gene was associated (*p* = 2.00e-07) with variance in anxiety symptoms. See Supplementary Information (**Figures S12**-**S16**) and **Table S6** for the top genes per phenotype.

The MAGMA competitive test of gene-sets (methods) identified nine significant associations after Bonferroni correction for multiple testing (Table 2). Two gene-sets were significantly associated with variance in depression symptoms, two with neuroticism, three with PLE, and two with autistic traits, one in adult and child samples each. See Supplementary Information **Table S7** for top gene-sets per phenotype, **Tables S8-S11** for details of significant gene-sets and **Table S12** f for biological annotations for all genome-wide significant results.

We estimated the SNP-heritability of MZ differences using LDSC (v.1.0.1)^19^ (see methods). The SNP-heritabilty estimate of environmental sensitivity to ADHD symptoms in the adolescent samples was 0.18 (se = 0.11); the estimate in the child samples was 0.04 (se = 0.06). The SNP-heritability estimate for environmental sensitivity to adult autistic traits was 0.09 (se = 0.15), and for depression symptoms, it was 0.03 (se = 0.09 in children and se = 0.06 in adults). All estimates, including those from the remaining phenotypes were consistent with zero (**Table S13**). We could not estimate the genetic correlation between all phenotypes because the SNP-heritabilities were too low and imprecise, except for ADHD child and adolescent symptoms (*rg*=0.82, se = 0.56, *p* = 0.15).

**Figure 4:**
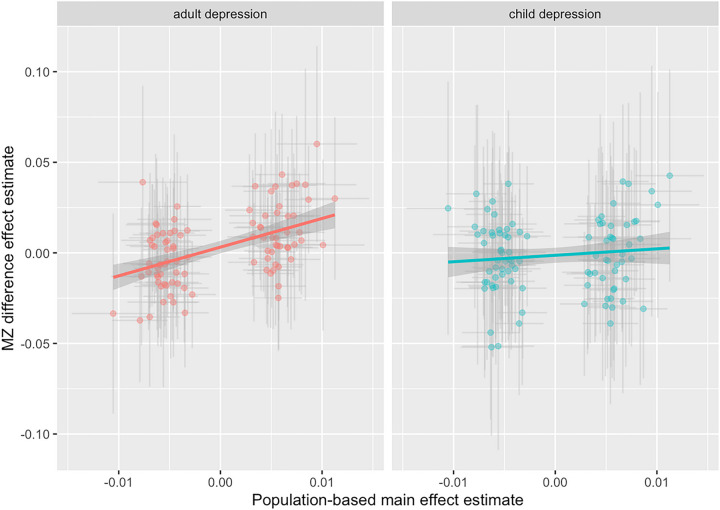
Two-sample Mendelian randomization estimate of the effect of genetic liability to depression on variance in depression (mean- variance relationship) Notes: Mendelian randomization estimate of the effect of genetic liability to depression from population studies (x-axis) on variance in depression scores (y-axis), using 102 variants discovered to associate with depression incidence ^41^. Genetic liability to depression increased variability in depression outcomes, which was attenuated to the null in childhood (interaction p-value=0.008).

It has been previously speculated that environmental sensitivity may relate to polygenic liability rather than single loci due to the environment interacting with a polygenic biological component^20^. We used Mendelian randomisation to estimate the influence of genetic liability of psychological phenotypes on their environmental sensitivity (see Methods). We found a strong effect for depression (beta = 0.84, se = 0.26, *p* = 0.002). We also ran the analyses separately for our child and adult samples (Fig. 4). The signal for depression was driven by analyses in adults (beta = 1.58, se = 0.29, *p* = 5e-7), with the childhood association was attenuated (beta = 0.35, se = 0.35, *p* = 0.36), these estimates differed (interaction p-value = 0.008). There was little evidence for heterogeneity of effect estimates across depression liability instruments. Other examined phenotypes did not exhibit an influence of liability on environmental sensitivity after correcting for multiple testing (**Table S14**). We found little evidence that mean body mass index levels and years of schooling affected phenotypic variance. Since variance QTL effects could be biased by main effects, we conducted simulations to evaluate how sensitive the analytical approach was to the MR results being driven by this bias (**Supplementary Information section 7**). We found that the MZ difference model can be liable to this problem under some phenotype normalisation approaches but that this is less likely to be driving the results in this study because we normalised by inverse-rank transformation (see **Figure S17**).

**Figure 5:**
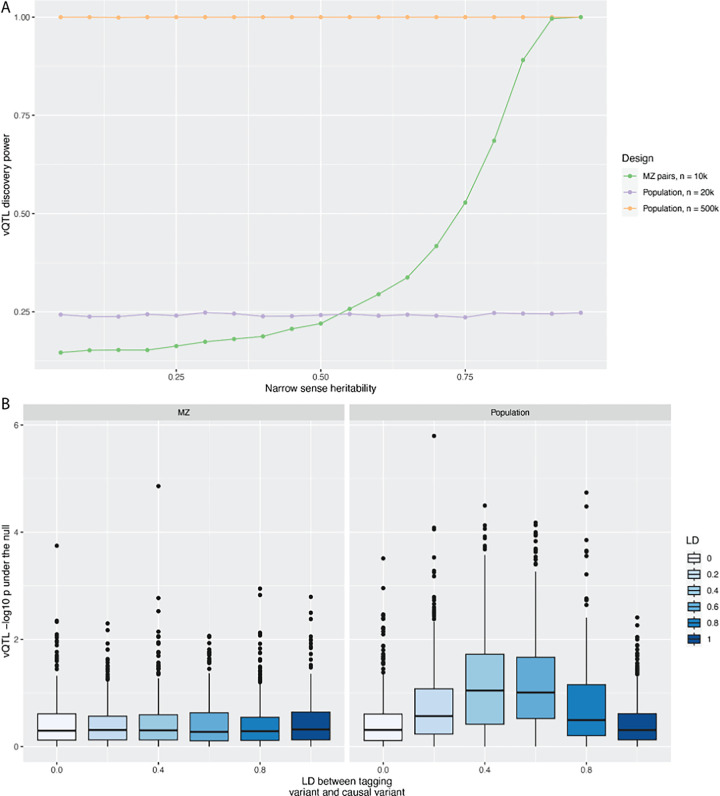
MZ differences approach complements population-based vQTL detection. **A**. Power comparison between the MZ differences approach and population-based approach (using the DRM method) for detecting vQTLs. The y-axis shows the fraction of simulations for which a true vQTL was detected at p < 0.05, and the x-axis shows the narrow sense heritability used in the simulation. The sample size for the MZ pairs is compared against an equal number of unrelated individuals in the population (20k) or a typical sample size in modern GWAS (500k). The MZ difference approach increases power as narrow sense heritability increases. **B.** False discovery rates (y-axis) due to incomplete LD with a tagging causal variant (x-axis), compared between the MZ difference approach (left box) and the population-based approach (right box). The presence of an additive causal variant tagging the tested SNP leads to elevated false discovery rates in the population approach but not in the MZ difference approach.

We used simulations to investigate the properties of the MZ-based variance loci detection methods and compared them to population-based vQTL methods. First, we found that the MZ-based methods have the greatest power when the narrow sense heritability is highest (Fig. 5A) and the residual variance is lowest, and therefore, when variance effects explain the larger fraction of the difference between MZ pairs, as suggested previously ^10^. Second, we found that for moderate heritability, the MZ difference approach has substantially greater statistical power than the population-based approach when sample sizes are equal (i.e., 10,000 MZ twin pairs using the MZ-based method versus 20k unrelated individuals using population-based vQTL method). However, in practice, the number of population-based samples generally available drastically outstrips the number of MZ samples available. When using a more realistic sample size (e.g., 10k MZ twin pairs versus 500k population samples), the MZ difference approach only achieves similar power to the population approach when narrow sense heritability values are very high (e.g., > 0.9). It has recently been shown that a variant tested for interaction can have inflated test statistics when in linkage disequilibrium with a strong additive effect^8^, and we investigated if that mechanism can also adversely impact MZ-based estimates. Our simulations show that this problem is substantially exacerbated through population-based vQTL methods compared to direct interaction tests, but the MZ-based approach is robust to this bias (Fig. 5B).

Several genome-wide significant results were notable, including our finding that *PTCH1* gene was associated with variation in depression symptoms, as this gene has previously been reported to be associated with depression-related phenotypes, including neuroticism^21,22^ , anxiety23,24, depression symptoms21, feeling emotionally hurt25 and sensitivity to environmental stress and adversity25. *C15orf38* gene (also known as *ARPIN-AP3S2*) was associated with variance in anxiety symptoms in our child samples and has previously been associated with type 2 diabetes in adults22,26 and corticotropin-releasing factor protein levels27, which are involved in regulating anxiety, mood, eating, and inflammation^28^. Hypoglycaemia symptoms in Type 2 diabetes include rapid heartbeat, sweating, and nervousness, all of which are physical sensations associated with anxiety. It is possible that certain variants in this gene impact sensitivity to the effects of diet and stressors that are involved in variability in insulin^29^, unpleasant physical sensations of which may be contextualised and made sense of as worries and anxieties (e.g., two-factor model of emotions^30^).

For autistic traits, the identified gene-set included genes involved in tissue morphogenesis and healing, which regulate response to transforming growth factor beta (TGFβ-1) levels and are involved in tissue repair pathways^31^. Growth factors serve important roles in neurodevelopment, immune function, and development of the central nervous system, and there is evidence that autism is associated with TGFβ-1 and other growth factor genes^32,33,34^. For psychotic-like experiences, the gene-sets related to the regulation of dopamine and catecholamine uptake. Our findings are supported by catecholamine’s involvement in stress response and the hypothesised role of dopamine systems dysregulation in the aetiology of psychosis^35^. Since gene-environment interactions have been implicated in variations in psychotic-like experiences^36^, the association between the biological processes implicated herein and variability in psychotic experiences may partly be under the influence of the environment.

We also estimated, for the first time, the SNP-heritability of environmental sensitivity (adolescent ADHD h^2^ = 0.18, se = 0.11 and child h^2^ = 0.04, se = 0.06 adult autistic traits h^2^ = 0.09, se = 0.15; depression h^2^ = 0.03, se = 0.09), but the estimates were consistent with zero. We also showed variants that affect mean levels of depression and anxiety influence these phenotypes’ variance. Several population-based methods for vQTL analysis are known to be susceptible to bias due to mean effects. By contrast, in principle, the MZ difference design is protected from this problem. Therefore, our study provides independent evidence that mean effects can influence variance.

The main strengths of our study were using the MZ differences design to investigate the genetics of environmental sensitivity in the largest sample of MZ twins and in a wide range of psychological phenotypes from a *developmental perspective*. The main limitation was limited statistical power to detect small genetic effects on variance. Further, as with all empirical analyses, our inferences depend on specific assumptions, which may not hold. First, we have taken the MZ differences score to reflect *genuine phenotypic variability*. MZ differences, however, also reflect measurement errors that are difficult to separate from genuine phenotypic differences, and it is unclear if MZ differences are stable across time. Future projects should investigate this approach with other low-measurement-error phenotypes, such as height, and consider a longitudinal design to assess the stability of these differences. Second, it is challenging to determine which mechanisms explain phenotypic variance: genetic sensitivity to the environment, epigenetic processes such as DNA methylation, imprinting, chorionicity, and skewed x-inactivation (for female MZ twins) also contribute to MZ phenotypic differences ^13^,^37^. Also, since our samples were all of European ancestry, our findings may not be generalisable to non-European ancestry.

In summary, we identified novel genetic factors associated with phenotypic variability. Our study illustrates the importance of large meta-analyses of genotyped MZ twins samples as a new source for discovering and understanding the genetics of phenotypic variance.

## Online Methods

### Study design

Figure 3 shows the flow chart for the current study. Twin cohorts that were part of the *within family consortium*, as well as additional cohorts, were invited to take part in the current study if they satisfied **all** of the following criteria:

- had at least 100 pairs of MZ twins
- one or both twins must be genotyped,
- imputed genotype data is available (e.g. 1000 Genomes or HRC),
- both twins must have complete data for one or more phenotypes. Imputation of missing data for incomplete pairs is not recommended.
- both twins must have complete covariate data (age, sex, and principal components for the genotyped twin)
- samples are of European ancestry.

The analysis plan and pipeline were written and pre-specificied (here) and shared with interested cohorts to conduct GWAS of MZ differences on their available phenotypes. The results were to be uploaded to a designated repository.

Some participating studies had collected data across the lifespan, which comprised repeated measures for certain phenotypes. It was therefore possible to explore genetic associations in the context of development by conducting developmentally stratified analyses. The developmental groups were defined as childhood (5–12 years old), adolescence (13–18 years old), and adulthood (> 18 years old). Cohorts conducted GWAS analyses separately for each developmental stage if data were available. In the end, for anxiety symptoms, depression symptoms and autistic traits, data GWAS data were available for children and adults. For ADHD symptoms, GWAS were available for children and adolescents. For well-being and neuroticism, samples consisted of adults and for psychotic-like experiences the samples were adolescents.

Two sets of meta-analyses were then conducted using the GWAS results: developmentally-informed, whereby GWAS results across studies were grouped according to the developmental stage of the sample and meta-analyses were conducted per phenotype (e.g., depression–child, depression-adult); and developmentally-agnostic, whereby a meta-analysis was conducted for each phenotype, using the largest sample from each study, regardless of developmental stage (e.g., depression-largest, anxiety-largest). This ensured maximum power for meta-analysis per phenotype via the largest N (see Supplementary Information **section 1.1 for more details on study design**).

### Samples

Samples included MZ twin pairs from cohort studies or twin registries in Australia and Europe (see **Supplementary Information Section 1.2.** and **Table S1** for details of participating studies). Informed consent and ethical approvals were obtained for all participating cohorts (see supplementary information). Table 1 shows the total sample sizes per phenotype and across developmental groups.

### Phenotypes

The MZ differences method requires continuous or categorical non-binary phenotypic data to calculate variance. Therefore, we used mean symptom scores instead of case/control diagnosis, and preference was for continuous measures. Various instruments have been developed to assess psychological phenotypes, which were reflected in the participating studies. The scales differed in the number of items included, the types of symptoms assessed, and the informant source. If multiple rating scales of a phenotype were available, studies were asked to select the scale with the most items (tapping most symptom domains). Scales were coded so that higher values represented higher symptom levels. The absolute phenotypic differences (APD) were obtained for each MZ pair. Using linear regression, APD was corrected for age, sex, ten genetic principal components, and any study-specific covariates. The residuals were standardised and rank-transformed to be used as the phenotype in GWAS. Below is a brief description of each phenotype (see **Supplementary Information Section 1.1** for details).

**Attention-deficit hyperactivity disorder (ADHD)** is a childhood-onset neurodevelopmental disorder of attention, activity and impulsivity. ADHD symptoms commonly persist into adulthood. ADHD symptoms are typically measured continuously using rating scales, often with separate scales for attention problems and Hyperactivity/impulsivity, which can be summed into a total score of ADHD symptoms.

**Anxiety** is heterogeneous, with clinical diagnoses consisting of specific anxiety disorders (e.g. phobias, post-traumatic stress disorder, social anxiety disorder) and generalised anxiety disorder (GAD). We were interested in generalised anxiety symptoms, usually measured via self-report, reflected in a total score of anxiety symptoms.

**Autism spectrum disorders (ASD)** are neurodevelopmental disorders broadly reflecting difficulties in social interaction and verbal communication, and repetitive behaviours. Symptoms typically emerge in early childhood, and assessment is carried out via questionnaires and or interviews. The continuous scores reflect presence/extent of autistic traits rather than ASD diagnosis.

**Depression** is heterogeneous, with many clinical presentations. The diagnosis requires a distinct change of mood, characterised by sadness or irritability, accompanied by psychophysiological changes, such as disturbances in sleep and appetite and loss of the ability to experience pleasure. The phenotypic scores for depression reflect presence of any of these symptoms rather than diagnosis of major depression.

**Neuroticism** is a personality domain and refers to a lack of emotional stability, stress vulnerability, the tendency to experience intense negative emotions, affects, and cognitions, and impulsive behaviours under emotional strain. Neuroticism is considered a risk factor for anxiety and depression.

**Psychotic-like experiences (PLE)** include a sub-clinical threshold of symptoms related to psychosis/schizophrenia disorders, such as persecutory ideation or perceptual abnormalities, prevalent in the community and non-clinical samples. PLEs are screened using self/other report questionnaires or interviews that cover some or all of these domains: paranoia, hallucinations, cognitive disorganisation, anhedonia, and negative symptoms.

**Wellbeing** includes both hedonic and eudaimonic well-being, assessed typically via questionnaires, indexing an individual’s subjective sense of wellness, such as reporting satisfaction with one’s life or being hopeful and optimistic about it. The data from participating studies mainly related to subjective well-being (e.g., life satisfaction). We preferred to use data where well-being has been measured using a battery of questions. If data were only available from a single question reported on a Likert scale, the response variable was treated as a continuous scale.

### Genotypes

Studies were required to have genotype data from all 22 autosomes imputed to either the 1000 genomes reference panel (preferably phase 3) or the Haplotype Reference Consortium (HRC). Almost all contributing studies had already participated in a related project from the *Within Family Consortium*^7^, and used the same protocol in the automated scripts for genetic data preparation and quality control (QC) procedures before GWAS analysis. Minimum quality control requirements at the study level included filtering SNPs for imputation quality > 0.3 for HapMap imputed data and > 0.5 for 1000G or HRC data, call rate > 95%, and minor allele frequency (MAF) > 1%. Studies also removed one pair randomly when there were two MZ pairs with kinship > 0.1. Study-level genotyping and QC information are included in Supplementary Information (**Gentoyping_AllStudies.xlsx**.)

### Analyses

#### Simulations

We investigated the statistical properties of MZ differences GWAS method using simulations. Family-based design, such as the MZ differences method, complements population-based vQTL methods in three ways: a) statistical power, b) robustness to bias due to additive effects, and c) provides an alternative identification strategy for triangulation^38^. To simulate variance QTL, we used the following data-generating model.

$$y_{i,t}=\alpha +\beta_{1,j}G_{i}+z_{i}+v_{i,t}+e_{i,t}$$

where α is an intercept term, $\beta_{1,j}$ is the additive effect of SNP j, $G_{i}$ is the genotype value for MZ pair i={1,…,N}, twin t={A,B}, at SNPj={1,…J} such that

G∼Binom(2,p)

where p is the allele frequency. $z_{i}$ is the remaining polygenic risk defined as

$$z_{i}∼N\left(0,\sigma_{g}^{2}-2p(1-p)\beta_{1}^{2}\right)$$

where ${\sigma^{2}}_{g}$ is the genetic variance of the trait, and $v_{i}$ is the SNPs’ combined influence on phenotypic variance. The SNP inflates the variance by $\beta_{2}$.

$$v_{i,t}∼N\left(0,\beta_{2}G_{i}\right)$$

and $e_{i}$ is the residual variance defined as

$$e_{i,t}∼N\left(0,1-{\sigma^{2}}_{g}-{\sigma^{2}}_{v}\right)$$

We estimated vQTL effects using unrelated individuals and the deviation regression model (DRM) from Marderstein and colleagues^39^. For a pair (A and B) of MZsG is fixed and $y_{i,A}$ and $y_{i,B}$ were generated identically but with independent error terms (i.e. $v_{i,t}+e_{i,t}$ ). We estimated vQTL effects using MZs and the following MZ difference model:

$$\left|y_{i,A}-y_{i,B}\right|=\beta_{2}G_{i}+\epsilon_{i}$$

We investigated the power of each method by generating vQTL effects $\left(\beta_{2}\right)$ calibrated to have 80% statistical power for the DRM method in 500,000 unrelated individuals (with parameters p=0.3 and $\beta_{1}=0$ ). We then estimated how power of the MZ difference approach varies for these parameters across a range of narrow sense heritability values ${\sigma^{2}}_{g}=h^{2}$. We calculated the power for detecting a vQTL at genome-wide significance levels, by drawing 1000 replications and identifying the fraction of simulations that had p<5×10^−8^).

We estimated the false discovery rate for vQTL in the presence of tagging additive loci, following the approach outlined by Hemani and colleagues^8^. Briefly, the data generating model described above is simulated with an additive effect of $\beta_{1}=0.1$ generated, but the vQTL test is performed at a second locus $G^{*}$ which is generated to be in linkage disequilibrium with G. The simulations are then performed with no vQTL effect $\left(\beta_{2}=0\right)$ and varying LD between G and $G^{*}$ between LD $r^{2}=0,\ldots ,1,h^{2}=0.5,\;p=0.1$ and 500 repeats were performed for each parameter combination.

#### GWAS model

In our primary analysis, we estimated the association of absolute phenotypic difference between MZ twins (residualised for age, sex and PCs, then standardised and rank-transformed) and the genetic marker using linear regression for each SNP j:

$$\left|y_{i,A}-y_{i,B}\right|∼\beta_{2,j}G_{ij}+\epsilon_{i,j}$$

We constructed two further models for sensitivity analyses: Model 2, where the within-twin mean of the phenotype was a covariate in the regression, and Model 3, which differed from our primary model by not adjusting for PCs when constructing the phenotype. Model 2 was constructed to examine if adjusting for within-twin mean in the GWAS model would significantly impact the SNP-associations, which would be the case if the MZ differences largely reflected mean differences. However, this also risks over-correcting, especially for vQTLs, which affect both the mean and variance of a phenotype, which was indicated here by small to moderate (r∼0.3to0.6) positive correlation between MZ phenotypic mean and MZ phenotypic differences in our sample and as previously proposed ^40^. Model 3 was constructed since some participating studies were likely to be very small (< 300). Including 10 PCs for all studies might have been overly conservative, leading to inflation of p-values.

We used the Sign-test to assess whether Model 2 and Model 3 results were similar to Model 1, as indicated by the correlation between betas, p-values and direction of effect. The results indicated Model 2 and 3 were not significantly different to Model 1 (**Table S3a-S3c**). Therefore, we considered Model 1 to be the most parsimonious: lower number of parameters than, but similar results to Model 2, while also correcting for population stratification confounding. The remaining analyses were therefore conducted using Model 1 results only.

##### QC procedure:

Study-level GWAS results (**Figures S2-S7**) were QC’d using EasyQC (v. 23.8)^16^. Variants with missing BETA, SE, P or INFO scores and SNPs with MAF < 0.01, and INFO score < 0.5 were removed. Cptid (chr:bp:A1:A2) were created, and alleles, effects and frequencies were checked in all GWAS results and harmonised according to their respective reference panel (1000 G Phase 3 version 5 or Haplotype Reference Consortium). SNPs with mismatching alleles to the reference panel were removed. Indels, monomorphic SNPs and duplicate SNPs that could also be tri-allelic (same bp position with different alleles) were removed, retaining only the SNP with the largest sample. Manhattan and QQ plots were obtained, and lambda-median values were inspected for p-value inflation (see **Figures S2-S6** and **Table S2** for more details).

### GWAS meta-analysis

METAL (2011 release)^17^ was used to conduct inverse-variance weighted fixed effect meta-analysis across studies, per phenotype. First, a meta-analysis was conducted by selecting the GWAS result with the largest sample from each study, regardless of the developmental stage. Another set of GWAS meta-analyses were then conducted in developmentally-stratified samples for depression (child, adult), anxiety (child, adult), ADHD (child, adolescent), and autistic traits (child, adult) phenotypes (see **Table S4**). Cptid were mapped into rs ids from 1000 genome phase 3 version 5 European panel. SNP2Gene function in the FUMA web application (v1.5.0)^18^ was used to annotate GWAS SNPs and identify independent significant SNPs (SNPs which are in LD of the lead SNP at r^2^ = 0.1; lead SNPs are those in LD with any of independent significant SNPs with r^2^ > 0.6; **Table S5**), and for producing regional plots and QQ and Manhattan plots (see **Figures S7 to S11**).

#### Gene-based and gene-set analyses:

MAGMA (v1.08) in FUMA web application (v1.5.2)^18^ was used to annotate GWAS SNPs and conduct gene-based and gene-set analyses. Meta-analysed GWAS results were filtered to include only SNPs available in at least 50% of studies. SNPs were annotated to ensemble v.92 protein-coding genes for gene-based analyses using default parameters (SNP-wise model for gene test). A competitive test was conducted for gene-set analyses using default gene sets in FUMA from MsigDB v7.0, totalling 15496 gene sets (Curated gene sets:5500, GO terms:9996). Curated gene sets contain 9 data resources, including KEGG, Reactome and BioCarta. GO terms consist of three categories: biological processes (bp), cellular components (cc) and molecular functions (mf). The MHC region was excluded from all annotations (See **Table S5** for details).

### Heritability analysis

SNP heritability estimates per phenotype were obtained using LDSC (v. 1.0.1)^19^. The European 1000 Genomes LD scores generated by the authors of LDSC were used, and SNPs for heritability analyses were merged with a set of ~ 1.2 million high-quality SNPs defined by the authors of LDSC^19^.

### Developmentally stratified analyses

For ADHD symptoms, data were available for children (5–12 years old) and adolescents (13–18 years old), whereas for anxiety, depression and autistic traits, data were available for children and adults (> 18 years old). The stratified analyses included a meta-analysis of GWAS results separately for children, adolescents, or adult samples for these phenotypes, followed by gene-based and gene-set and heritability analyses, using the same criterion as the largest non-stratified sample.

#### Mendelian Randomisation analysis:

We used a two-sample summary data Mendelian randomization to estimate the influence of genetic liability of psychological phenotypes on their environmental sensitivity. Mendelian randomization uses genetic variants as instrumental variables for the exposure of interest. Three assumptions define instrumental variables. First, relevance: the instrument must be associated with the exposure. Second, independence, there must be no uncontrolled confounders of the instrument-outcome association, and third, the exclusion restriction, that the instruments only affect the outcome via the exposure of interest. We selected 102 independent (LD = 10,000kb, R^2^ = 0.001) genetic variants associated with depression in Howard, et al. ^41^ as instruments for genetic liability to depression. We harmonized the effects by effect allele, chromosome and position on build HG19. We used the inverse variance weighted estimator to estimate the effect of genetic liability to depression on phenotypic variability using the TwoSampleMR package^42^. The variants strongly associated with depression and there was minimal overlap between our samples and those used in the GWAS to select variants. Therefore, weak instrument bias is unlikely^43^. We followed the same procedure for the other phenotypes but with a different number of SNPs (see **Table S14**). Because depression is strongly genetically correlated with anxiety, but there are no well-powered GWAS for anxiety, we performed a similar analysis for variance in anxiety but using the 102 variants for depression. Here, the main effects for anxiety at each of the 102 variants were obtained from a GWAS in UK Biobank for self-reported anxiety measures^44^. Finally, we also tested educational attainment^45^and body mass index. GWAS summary statistics for main effects were obtained from OpenGWAS^46^. We reported this analysis using the STROBE-MR checklist^47^.

## Figures and Tables

**Table 1:** Descriptives of the samples in the current study

Phenotype	Sample	N studies	N of MZ twins*	mean age	mean rMZ
**ADHD symptoms**	Largest	4	13738	9.54	0.71
	Child	4	13738	9.54	0.71
	Adolescents	4	7840	20.28	0.58
**Anxiety symptoms**	Largest	5	12354	20.40	0.52
	Child	3	10494	10.01	0.54
	Adult	5	7932	33.35	0.46
**Autistic traits**	Largest	4	14152	15.79	0.63
	Child	3	13130	10.22	0.69
	Adult	3	6050	22.53	0.63
**Depression symptoms**	Largest	11	21792	43.23	0.41
	Child	3	10510	10.05	0.51
	Adult	11	18074	43.62	0.38
**Neuroticism**	Largest	4	8900	35.05	0.35
**Psychotic-like experiences**	Largest	2	3636	15.91	0.46
**Wellbeing**	Largest	9	13740	41.30	0.31

Notes: Largest: largest available sample, obtained by selecting the largest sample from each study, irrespective of age group. Child: data from studies where participants were aged 5–12 years old; Adolescent: data from studies where participants were aged 13–18 years old; Adult: data from studies where participants were aged > 18 years old.

* GWAS N is halved as only genotype data from one twin from each pair is used for GWAS; mean rMZ = average Monozygotic twin correlation across studies; PLE = psychotic-like experiences.

**Table 2:** Genome-wide significant gene-based and gene-set results

GWAS meta-analyses												
Phenotype	Sample	SNP	CHR	Position	Gene	A1	EAF	Beta	SE	P	N	Effect across studies
Anxiety	Adult	rs60358762	13	99411217	*SLC15A1*	A	0.03	0.44	0.1	5.07E-09	3033	+++??
Wellbeing	Adult	rs2940988	4	37586376	*C4orf19*	T	0.88	0.16	0.03	9.93E-09	6464	++?++−+++
Gene-based analysis								
Phenotype	Sample	Gene	Chr	NSNPS	NPARAM	N	ZSTAT	P
Anxiety	Largest	*C15orf38*	15	28	7	5265	5.08	2.00E-07^a^
Depression	Largest	*PTCH1*	9	130	12	10166	4.63	1.80E-06^b^
Gene-set analysis								
Pheno	Sample	Gene Set	Ngenes	Beta	SD	SE	P	Pbon
Autistic traits	Child	Plasari tgfb1 targets 1hr down regulated	5	1.68	0.03	0.34	6.00E-07	0.01
	Adult	Regulation of protein localization to cilium	7	1.43	0.03	0.26	1.00E-08	0.0002
Depression	Child	Proteasome regulatory particle	19	0.79	0.03	0.17	1.41E-06	0.02
		Proteasome degradation	50	0.52	0.03	0.11	2.48E-06	0.04
Neuro	Adult	Gemini of coiled bodies	9	1.01	0.02	0.20	2.00E-07	0.003
		Negative regulation of vasculature development	92	0.38	0.03	0.08	5.97E-07	0.01
Psychotic-like experiences	Adolescent	regulation of dopamine uptake involved in synaptic transmission	8	1.37	0.03	0.28	5.00E-07	0.01
		catecholamine uptake involved in synaptic transmission	11	1.23	0.03	0.26	9.55E-07	0.01
		extrinsic component of endoplasmic reticulum membrane	5	1.63	0.03	0.35	1.59E-06	0.02

Notes: Largest: largest available sample, obtained by selecting the largest sample from each study, irrespective of age group. Child: data from studies where participants were aged 5–12 years old; Adolescent: data from studies where participants were aged 13–18 years old; Adult: data from studies where participants were aged > 18 years old. ?: SNP missing in the study

a b Bonferroni-corrected p-value significance threshold were, a: P = 0.05/18535 = 2.698e-6 and b: P = 0.05/18624 = 2.685e-6

## Data Availability

Meta-analysed GWAS summary statistics will be made publically available following publication. Data from individual studies are not publicly available and are subject to strict access control, since the consent given by the participants does not allow for data storage on an individual level in repositories or journals. Access to these data requires specific approval from the relevant data access committees for each cohort.
